# The role of ball mass, surface, and contact dynamics in mechanochemical reactions

**DOI:** 10.1039/d5mr00146c

**Published:** 2026-01-28

**Authors:** Marisol F. Rappen, Justus Mäder, Tino Schwemin, Sven Grätz, Lars Borchardt

**Affiliations:** a Ruhr-Universität Bochum Universitätsstraße 150 44801 Bochum Germany Lars.Borchardt@ruhr-uni-bochum.de

## Abstract

In this work, the influence of milling ball properties on energy transfer and mixing efficiency was systematically investigated by decoupling mass, surface area, and kinetic energy. To achieve this, hollow and solid balls of different sizes were employed, allowing independent variation of these parameters. Additionally, cylindrical and round-ended milling tools were additionally used to study the effects of surface geometry and contact dynamics. Yield normalization by energy input, ball mass, and surface area enables clearer correlation between individual ball characteristics and milling efficiency. This approach provides a more detailed understanding of how individual mechanical properties contribute to overall process performance.

## Abstract 2

This work studies how mass, surface area and contact dynamics affect mechanochemistry using hollow balls and cylindrical tools. Two model reactions are examined: the Finkelstein reaction and the direct mechanocatalysed Suzuki coupling.

## Graphical abstract

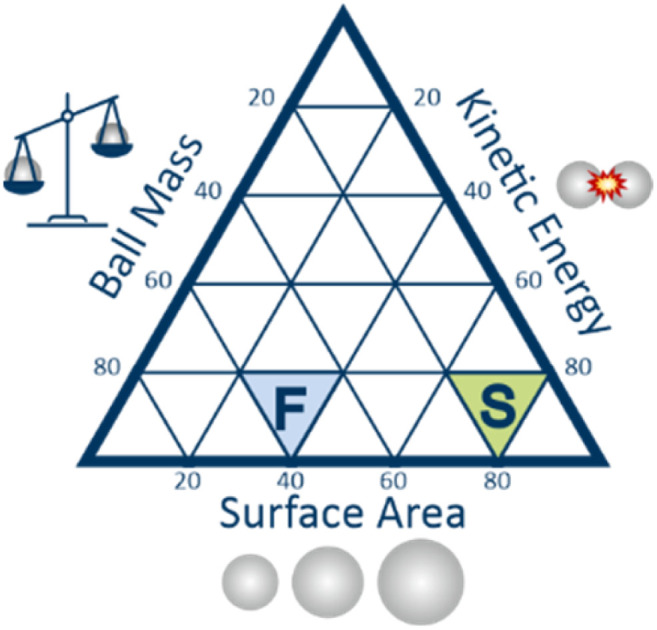

## Introduction

Mechanochemistry offers a fundamentally different approach to chemical synthesis by enabling reactions to proceed under solid-state conditions, often minimizing or even eliminating the need for solvents.^1^ Unlike conventional solution-based methods, this strategy provides a cleaner and more sustainable route, minimizing waste while maintaining strict control over reaction parameters.^2–5^ Such advantages have fuelled its growing adoption in areas ranging from the preparation of advanced materials to the development of pharmaceuticals and fine chemicals.^6–10^ A key strength of this methodology lies in its tunability: reaction outcomes can be systematically optimized through adjustments in operational factors such as milling frequency or reaction time. For numerous types of chemical transformations, it was shown, that increasing the energy input is beneficial for a successful product formation.^11,12^ Employing denser milling materials or raising the reaction frequency or temperature can therefore have a major impact on reactivity.^13^ However, higher energy input does not always lead to maximized yields.^14^ Moreover, discrepancies in yields often arise when, for example, the mill type is varied—since not only the energy input but also the type of mechanical forces differ.^15^

**Fig. 1:**
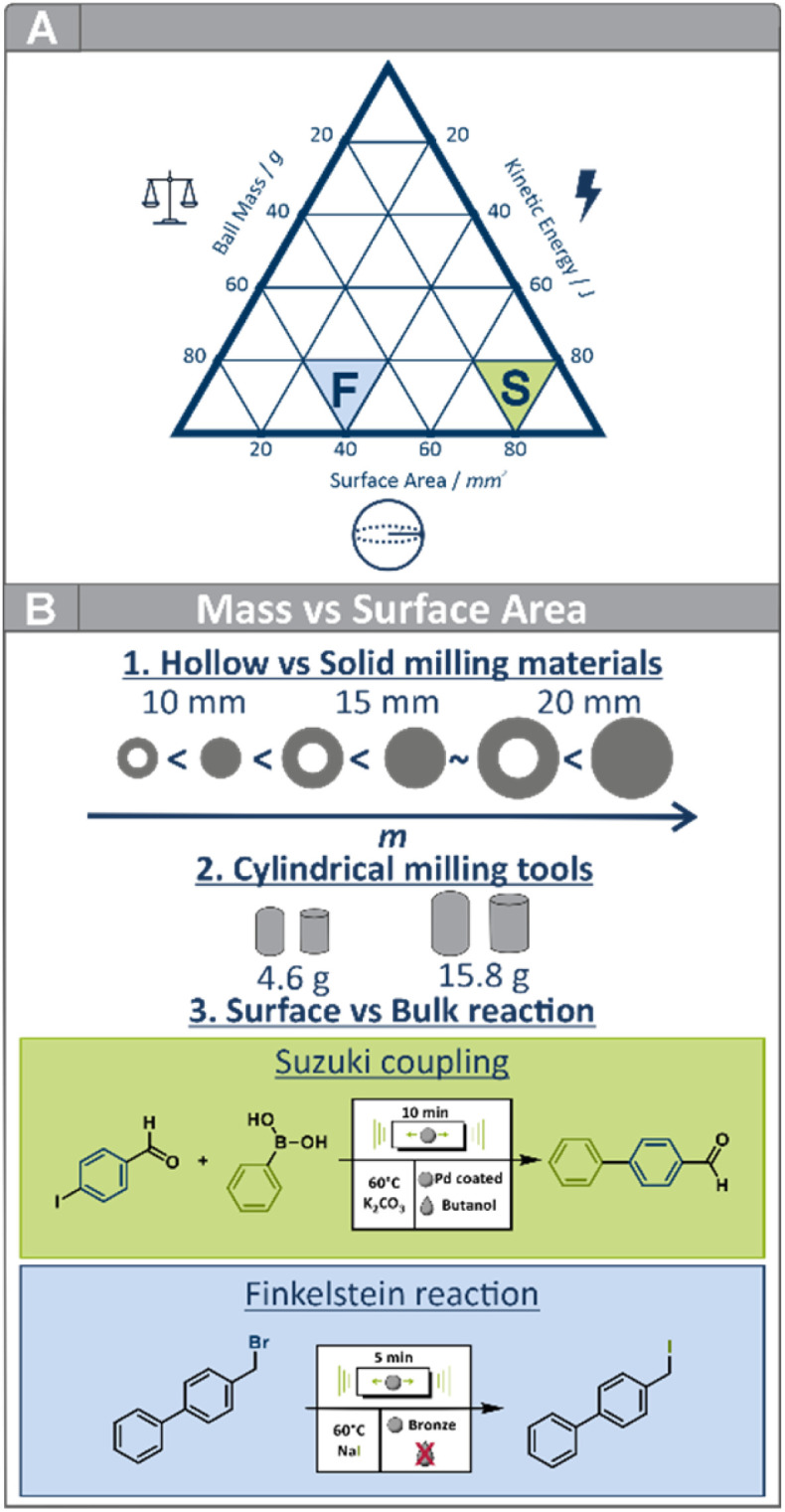
(A) Conceptual overview of this study and schematic visualization of the interplay of the key parameters mass, surface area, and energy input for the Finkelstein reaction (blue triangle, F) and the Suzuki coupling (green triangle, S). The figure serves as a conceptual illustration and does not imply quantitative relationships. (B) Schematic representation of the milling media used (solid, hollow). Reference reactions: the surface-dependent Suzuki coupling and the Finkelstein reaction occurring in the bulk.

Our previous work has already demonstrated the influence of milling ball trajectories on mechanochemical reactions, underscoring the importance of parameters such as the effective contact area, as well as the frequency, and duration of contact between the balls, the vessel wall, and thus the substrates caused by a change in ball motion.^16^ Further research in our group revealed distinct effects of single impact energies, cumulative energies, and the addition of Liquid-assisted grinding (LAG) and aging on distinct mechanochemical transformations.^3,17^ Both of these findings further highlight that mechanochemistry cannot be fully understood by considering total energy input alone (Fig. 1).^16^ Instead, energy transfer is intimately linked to factors such as ball velocity, ball mass, and the temperature generated inside the vessel.^18,19^

The milling tool's properties are therefore of high importance, whereby most studies specifically addressing the role of the milling ball typically rely on balls of different sizes.^19,20^ However, this approach inherently alters not only the mass but also the surface area of the milling ball. A common way to circumvent this issue is to use balls of identical size but different densities, which, however, also changes the material properties.^15^ Michalchuk *et al.* proposed an alternative approach by employing nylon balls with a lead core, demonstrating that both mass and surface area play a crucial role.^21^ To extend the findings of Michalchuk *et al.*, our approach further considers the effects of reaction temperature and aims to provide mechanistic insights into ball trajectories, thereby helping to further elucidate energy transfer in mechanochemical reactions.

Combining controlled near-isothermal conditions with insights into ball trajectories, kinetic energies and two mechanistically distinct reactions—one dominated by bulk processes and the other by surface interactions—this study offers a comprehensive understanding of how ball architecture governs mechanochemical reactivity (Fig. 1). Ultimately, this work contributes to a deeper understanding of energy transfer in mechanochemical processes, paving the way toward more rational design and optimization of reaction conditions.

## Results and discussion

### Prolog

Building on our investigation of ball motion and contact dynamics, we aimed to further explore how other ball characteristics contribute to mechanochemical processes. In particular, properties such as mass (*m*) and total surface area (*A*) of the milling ball are of interest, as both are closely linked to the energy transfer occurring inside the ball mill. However, these properties are inherently interdependent. For example, increasing the ball size simultaneously alters its mass and available surface area, thereby changing the single impact- and total kinetic energy introduced into the system. This close coupling makes it challenging to isolate the individual effects of mass, kinetic energy (*E*_kin_), and surface area—especially in cases such as direct mechanocatalysis, where reactions occur directly on the surface of the milling media.^22–26^ As a result, these effects cannot be investigated independently by simply using larger milling balls. To overcome this limitation, hollow milling balls with diameters of 10 mm, 15 mm and 20 mm made from bronze were employed in this study (Table 1). These allow for a reduction in ball mass while maintaining a catalytic surface area comparable to that of a solid metal ball of the same diameter.

**Table 1:** Properties of the differently sized hollow and solid milling balls, as well as cylinders used in the course of this work. Shown are the mass (*m*) in g, the total surface area (*A*) in mm^2^ and the kinetic energies (*E*_kin_) in mJ. The kinetic energy was experimentally determined in our previous work through the high-speed recordings (SI Chapter 4)

Milling tool	*m*/g	*A*/mm^2^	*E*_kin_/mJ^a^	*E*_kin_/mJ^b^
**10 mm ball**				
Hollow	2.6	314	12	—
Solid	4.6	314	40	—

**15 mm ball**				
Hollow	7.3	707	47	74
Solid	15.8	707	145	162

**20 mm ball**				
Hollow	15.1	1257	—	122
Solid	37.3	1257	—	355

**Cylinder** c				
Small flat end	4.6	397	35^d^	—
Small rounded	4.6	363	17^d^	—
Large flat end	15.8	905	38^d^	—
Large rounded	15.8	814	40^d^	—

a kinetic energy in the 14 mL vessel at 35 Hz.

b kinetic energy in the 40 mL vessel at 35 Hz.

c Detailed measurements are given in SI Chapter 1.1.

d The kinetic energies may deviate from the presented results, since it is not always possible to strike the center of the cylinders due to their asymmetry and irregular motion.

Further, two sizes of both regular cylinders as well as round-ended cylinders made from 1.3505 type steel were employed. These were designed to feature a similar mass as the 10 mm and 15 mm balls.

Table 1 shows different parameters of the differently sized hollow and solid milling balls used. Here, the mass in g, the surface area in mm^2^ and the kinetic energies in mJ obtained by the high-speed recordings are shown.^16^

All reactions were conducted in a MM500 mixer mill by *Retsch* at a milling frequency of 35 Hz at a temperature of approximately 60 °C. Previous measurements of the milling-induced temperature increase showed that the reaction mixture did not exceed 40 °C under the applied conditions. To ensure that no sample experienced significantly higher temperatures and to maintain comparable conditions across all experiments, the external heating was set to 60 °C. The temperature was controlled using heating jackets supplied by *Ihne & Tesch*. Two differently sized vessels made from PFA (perfluoroalkoxy polymer) or PA6 (Polyamide 6) were used (SI, Chapter 4.1.1). As reference reactions, the Finkelstein reaction of 4-(bromomethyl)-1,1′-biphenyl with sodium iodide (mainly occurring in the bulk) and the direct mechanocatalyzed Suzuki coupling of 4-iodobenzaldehyde with phenylboronic acid (taking place on the milling tool surface) were employed. These represent two fundamentally different reaction types, allowing the investigation of the different parameter's effects across distinct mechanistic pathways.

### Disentangling the effects of mass, surface, and energy

**Fig. 2:**
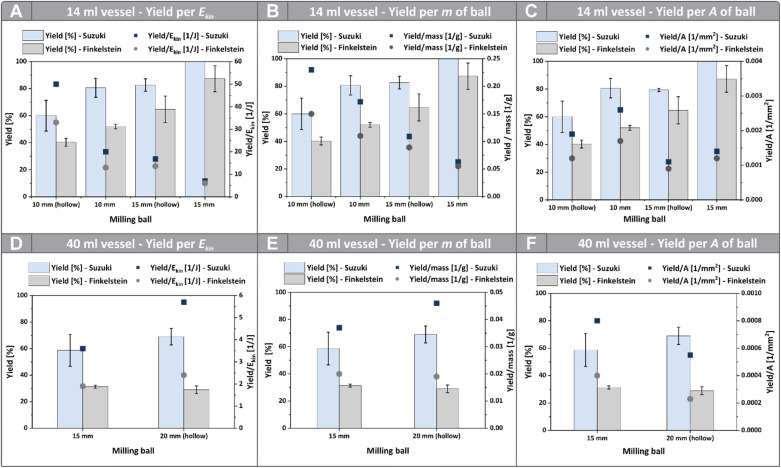
Reaction efficiencies for the Suzuki coupling and Finkelstein reaction using solid and hollow milling balls of different diameters. The same yield data are shown in each graph (A–C and D–F), normalized with respect to different parameters – mass, surface area, or energy input. (A–C) 10 mm and 15 mm solid and hollow balls; (D–F) 15 mm solid and 20 mm hollow balls. Efficiencies are expressed as (A and D) Yield/J, (B and E) Yield/g ball mass, and (C and F) Yield/mm^2^ surface area. Suzuki coupling: MM500 mixer mill (*Retsch*), 35 Hz, 10 min (Ø 10–15 mm; 14 mL vessel) or 5 min (Ø 15–20 mm; 40 mL vessel), 60 °C, 14 mL PA vessel or 40 mL PFA vessel; for the 14 mL vessel the reaction mixture contained K_2_CO_3_ (1.0 g, 7.3 mmol, 7.3 eq.), phenylboronic acid (122 mg, 1.0 mmol, 1.0 eq.), 4-iodobenzaldehyde (232 mg, 1.0 mmol, 1.0 eq.), and *n*-butanol (311 µL, *ƞ* = 0.15, 3.4 mmol, 3.4 eq.). For the 40 mL vessel the reaction mixture contained K_2_CO_3_ (2.9 g, 20.9 mmol, 20.9 eq.), phenylboronic acid (349 mg, 2.9 mmol, 2.9 eq.), 4-iodobenzaldehyde (663 mg, 2.9 mmol, 2.9 eq.), and *n*-butanol (889 µL, *ƞ* = 0.15, 9.7 mmol, 9.7 eq.). Yields were determined *via* HPLC. Finkelstein reaction: identical setup with 14 mL PA vessel containing 4-(bromomethyl)-1,1′-biphenyl (247 mg, 1.0 mmol, 1.0 eq.) and NaI (449 mg, 3.0 mmol, 3.0 eq.); milling time 5 min (Ø 10–15 mm). For the 40 mL vessel, the reaction was conducted in a PFA vessel containing 4-(bromomethyl)-1,1′-biphenyl (706 mg, 2.9 mmol, 2.9 eq.) and NaI (1.3 g, 8.6 mmol, 8.6 eq.); milling time of 2.5 min for the 40 mL vessel (Ø 15–20 mm). Yields were determined *via*^1^H NMR using benzyl benzoate as internal reference.

To systematically disentangle the effects of ball mass, surface area, and energy input on mechanochemical reactivity, we first examined the performance of custom-made hollow and solid milling balls of different diameters under near-isothermal conditions. This approach allows us to directly compare reaction outcomes while selectively varying individual ball properties. Fig. 2A–C present the yields of both the Finkelstein reaction and the Suzuki coupling using balls with a diameter of 10 mm and 15 mm inside a 14 mL vessel. To minimize artifacts from trajectory differences, we further conducted reactions in a larger vessel, where balls of different types follow more similar movement patterns. In our previous study, it was observed that the movement of 15 mm and 20 mm diameter milling balls in a 40 mL vessel is similar, with both following a figure-eight-shaped trajectory.^16^ Consequently, the contribution of ball movement to the observed trends is likely reduced when using the larger vessel, as the contact duration and frequency between the ball, vessel wall, and substrates are expected to be within a similar range. In contrast, in the 14 mL vessel, the 15 mm ball deviated from the figure-eight-shaped trajectory, making differences arising from the movement pattern more likely.

Here, balls with similar masses (difference of approximately 0.7 g) but changed surface areas (solid 15 mm ball *vs.* hollow 20 mm ball) were utilized. The results for the Suzuki coupling, as well as the Finkelstein reaction are shown in Fig. 2D–F. Here, the yields were further normalized to their respective masses, surface areas and kinetic energies.

For both reactions, the yields generated inside the smaller vessel increase with rising ball mass, kinetic energy, and surface area. A clear dependence on ball mass and therefore on the total kinetic energy is already evident when comparing solid and hollow balls of the same size (Fig. 2A–C). To gain further insights, normalizing yields by mass, energy, and surface area potentially helps clarifying the contributions of these parameters (Fig. 2A–C). Overall, the Finkelstein and Suzuki reactions show very similar trends once normalized.

It is important to note that ball mass and kinetic energy are closely linked, since changes in mass directly alter the energy transferred during collisions and friction events. This is reflected in the similar trends of yields normalized per gram of ball mass and per joule of kinetic energy. For both metrics, hollow balls outperform solid ones, yet this comparison alone does not identify the decisive factor controlling reactivity.

Normalizing the yields to the geometric surface area does provide clear insights, as the efficiencies of the solid balls are higher than those of hollow ones with the same surface area, further indicating a strong dependence on mass and kinetic energy (Fig. 2C). Despite the overall similarity in yields normalized to both mass and kinetic energy, noticeable deviations are observed for the solid 10 mm and hollow 15 mm milling balls. The difference in efficiency between these two balls becomes less pronounced when yields are expressed per joule of kinetic energy rather than per unit mass. Despite its 59% higher mass, the 15 mm ball exhibits a mean kinetic energy comparable to that of the smaller ball, emphasizing the dominant role of contact dynamics in mechanochemical transformations. This reduced kinetic energy arises from its less favourable trajectory and collision behaviour. Since ball trajectories critically influence contact frequency and duration, such dynamic effects may obscure potential surface-related contributions. Moreover, the reported surface areas represent the geometric total surface rather than the true contact area in collisions or during friction events.

The influence of the balls’ surface area therefore cannot be proofed by the results discussed so far. The larger 15 mm balls, for example, show lower efficiency than the 10 mm balls, complicating direct conclusions about this parameter (Fig. 2A–C).

For observing the influence of the surface area more precisely, the solid 15 mm and hollow 20 mm balls inside the larger vessel are of high interest, as these are similar in mass and movement pattern, but differ in their respective surface area. When comparing the results of the different balls used for the Suzuki coupling, it becomes evident that the yields, as well as the yields normalized to their respective masses and kinetic energies, are slightly higher for the larger ball (Fig. 2D and E). This suggests that the surface area—and therefore the actual contact area between the ball, the vessel wall, and the substrates—is of high importance, as the larger ball features a slightly lower mass and kinetic energy compared to the smaller 15 mm ball. Moreover, this observation further supports that the reaction takes place on the surface of the milling balls, in agreement with the previous findings.^22^ The yields normalized by the balls surface area, on the other hand, are not indicative.

For the Finkelstein reaction the yields, as well as the yields normalized by mass and kinetic energy, are similar. This potentially indicates that the influence of the balls surface area is somewhat less important for a reaction occurring predominantly in the bulk. In this case, the effects of kinetic energy and actual contact area appear to be more balanced, whereas in direct mechanocatalysis, the surface area plays a more dominant role.

In summary, the comparison of hollow and solid milling balls demonstrates that both mass and surface area contribute to mechanochemical reactivity. For the Suzuki coupling, the results indicate that both kinetic energy and surface area play significant roles, with the latter exerting an additional influence on the overall efficiency in direct mechanocatalysed reactions. In contrast, the Finkelstein reaction appears primarily governed by the effective energy input, showing only a minor sensitivity to surface-related effects. Overall, these findings highlight that the interplay between energy transfer and contact dynamics determines reaction outcomes in mechanochemical systems.

### Effect of geometry on energy transfer and reactivity

**Fig. 3:**
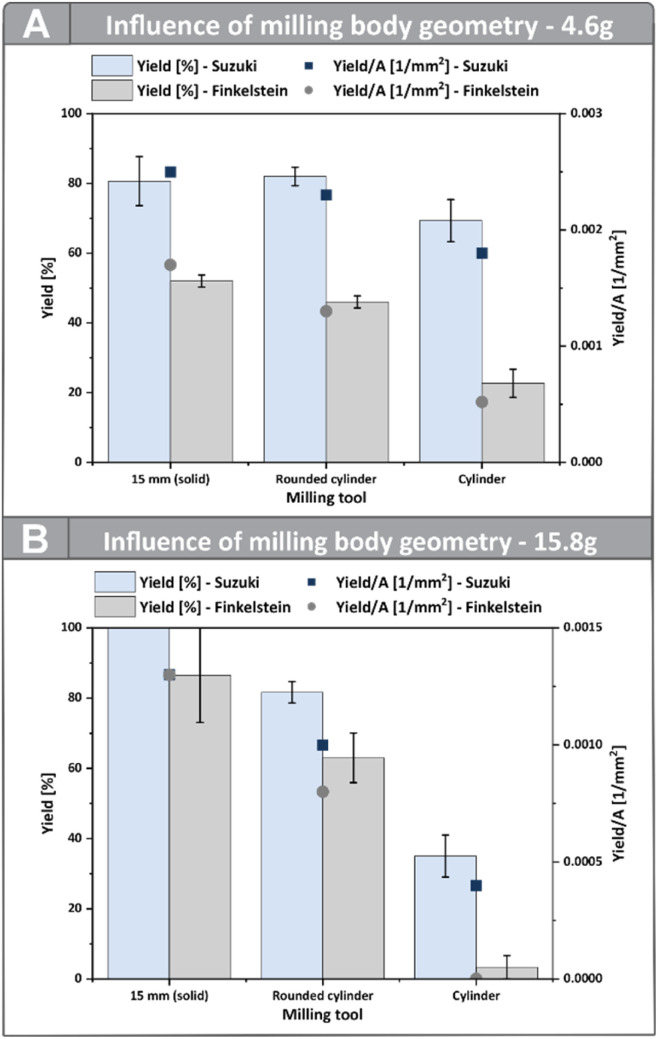
Comparison of yields obtained with cylindrical milling bodies of different shapes and milling balls of equal mass. (A) Direct mechanocatalysed Suzuki coupling; (B) Finkelstein reaction. Reactions were performed in an MM500 mixer mill (Retsch, 35 Hz, 60 °C) using 14 mL PA (Suzuki) or PFA (Finkelstein) vessels. Reaction mixtures contained K_2_CO_3_ (1.0 g, 7.3 mmol, 7.3 eq.), phenylboronic acid (122 mg, 1.0 mmol, 1.0 eq.), 4-iodobenzaldehyde (232 mg, 1.0 mmol, 1.0 eq.), *n*-butanol (311 µL, *ƞ* = 0.15, 3.4 mmol, 3.4 eq.) (Suzuki) and 4-(bromomethyl)-1,1′-biphenyl (247 mg, 1.0 mmol, 1.0 eq.) with NaI (449 mg, 3.0 mmol, 3.0 eq.) (Finkelstein); milling times were 10 min for the Suzuki reaction and 5 min for the Finkelstein reaction, respectively. Yields were determined *via*^1^H NMR using benzyl benzoate as internal standard (Finkelstein) or *via* HPLC (Suzuki).

To further investigate the influence of the milling tool surface area, as well as the contact dynamics, milling bodies with different geometries were utilized and compared to the milling balls used (Ø 10 mm and 15 mm). To this end, both regular cylinders and cylinders with rounded ends were employed, whereby for each geometry, bodies with masses of approximately 4.6 g and 15.8 g were utilized to allow for direct comparison with the previously used solid milling balls for both model reactions (Fig. 3A and B). For the direct mechanocatalysed Suzuki coupling, both types of cylinders with a mass of 4.6 g give similar results compared to the 10 mm ball used (Fig. 3A). For the milling tools with a mass of approximately 15.8 g the difference in yield is more pronounced. Here, the cylinder with flat ends differs from the 15 mm milling ball and the rounded cylinder by approximately 50%. The results for the samples prepared using the rounded cylinder are, however, in a similar range as those obtained with the milling balls. This can be explained by the fact that friction due to movement along the vessel walls or ends is more likely with the rounded cylinders tested, compared to the regular cylinders used. In the case of the Finkelstein reaction, both sizes of regular flat-ended cylinders performed inferior to the milling balls and the cylinders with rounded ends. This may be due to the reaction being more dependent on high-energy input. Flat-ended cylinders make less frequent and less effective contact with the vessel walls, likely resulting in lower energy transfer to the substrates compared to the other milling tools tested, despite its larger surface area.

When the milling tool efficiency is expressed as the yield per mm^2^ of total surface area, it becomes evident that the cylindrical tools generally perform worse than milling balls of comparable mass. For the smaller sized cylinders (Fig. 3A), the decrease in efficiency is, however, less dominant, as contact is still frequent compared to the larger cylinders used. This observation further emphasizes the critical role of contact dynamics in mechanochemical reactions, demonstrating that the actual contact area generated during impacts or frictional events—together with the contact duration and frequency—are decisive parameters alongside the kinetic energy.

## Conclusion

This work demonstrates that the relative contributions of mass, surface area, and kinetic energy in mechanochemical systems cannot be fully separated but can be partially resolved by controlled tool design. Hollow and solid milling bodies of identical material show that mass—and therefore impact energy—dominates reactivity in bulk reactions, while accessible surface area becomes critical for surface-catalyzed transformations. This represents a valuable extension of the work of Michalchuk *et al.*, demonstrating that while both mass and surface area are important parameters, their relative influence can vary depending on the specific mechanochemical system. Experiments with cylindrical tools reveal that apparent surface area alone is insufficient; contact dynamics and trajectory stability govern effective energy transfer. These results establish that mechanochemical efficiency is determined by the joint function of collision energy and contact mechanics rather than any single geometric or energetic descriptor.

Furthermore, this work strengthens the previously made assumption that the direct mechanocatalysed suzuki coupling is indeed taking place on the milling ball surface. Therefore, conducting a comparable experiment with milling balls of equal mass but different surface areas would provide a straightforward way to test whether a catalyzed reaction can truly be considered direct mechanocatalysis.

Beyond clarifying the interplay of these parameters, this study highlights the potential of tool geometry as a tunable design variable for optimizing mechanochemical reactions. Understanding how impact dynamics translate into reaction kinetics provides a foundation for rational process design and scale-up. Future integration with *in situ* diagnostics and computational models will be key to achieving fully predictive and adaptive mechanochemical systems. Overall, our study represents a valuable extension of the work of Michalchuk *et al.* by further resolving the influence of mass and surface area through the inclusion of near-isothermal conditions, two mechanistically distinct model reactions, and the consideration of contact dynamics.

## Author contributions

Marisol Fabienne Rappen: conceptualization, data curation, formal analysis, investigation, methodology, verification, visualization, writing – original draft. Justus Mäder: formal analysis, investigation, verification. Tino Schwemin: formal analysis, investigation, verification. Sven Grätz: conceptualization, data curation, funding acquisition, project administration, resources, supervision, writing – review & editing. Lars Borchardt: conceptualization, data curation, funding acquisition, project administration, resources, supervision, writing – review & editing.

## Conflicts of interest

There are no conflicts to declare.

## Supplementary Material

MR-003-D5MR00146C-s001

## Data Availability

The data supporting this article have been included as part of the supplementary information (SI). Supplementary information: details on the experimental set-up, the determination of the applied kinetic energies, and additional results for different milling tools that are not shown in the manuscript. See DOI: https://doi.org/10.1039/d5mr00146c.
