# Supplementary material for: The role of ball mass, surface, and contact dynamics in mechanochemical reactions
Source: RSC Mechanochem. 2026 Jan 28;3(3):357–62. doi: 10.1039/d5mr00146c (PMC12863241; doi:10.1039/d5mr00146c)
Supplement: MR-003-D5MR00146C-s001 [file MR-003-D5MR00146C-s001.pdf]

Supplementary Information (SI) for RSC Mechanochemistry.
This journal is © The Royal Society of Chemistry 2026

Supporting Information

The Role of Ball Mass, Surface, and Contact Dynamics in Mechanochemical Reactions

Marisol F. Rappen,[a] Justus Mäder,[a] Tino Schwemin,[a] Sven Grätz,[a] Lars Borchardt [a]

Table of contents

Supporting Information 1
1. Experimental section 3
1.1 Used equipment and characterisation techniques 3
2. Synthetic procedures 4
2.1 Direct mechanocatalysed Suzuki coupling 4
2.2 Finkelstein reaction 4
3. Product characterization 4
4. Kinetic energy calculations 6
5. Hollow vs solid milling balls – Role of mass and surface area 7
5.1 Suzuki coupling 7
5.1.1 Suzuki coupling PA vessel vs. PFA vessel 7
5.1.2 Suzuki Coupling – 14 mL vessel 7
5.1.3 Suzuki coupling – 40 mL vessel 8
5.2 Finkelstein reaction 10
References 11

1. Experimental section

1.1 Used equipment and characterisation techniques

General. All reagents were obtained from commercial suppliers at least in synthesis purity and used without further purification. The organic solvents utilised as LAG agents were purchased in analysis grade.[1] For all experiments conducted, a MM500 vario mixer mill (*Retsch*) was used. The milling vessels were made from PFA purchased from *Ammerflon*® GmbH and PA6 from *Technoplast.* Figure S1 shows the measurements of the milling vessels used.

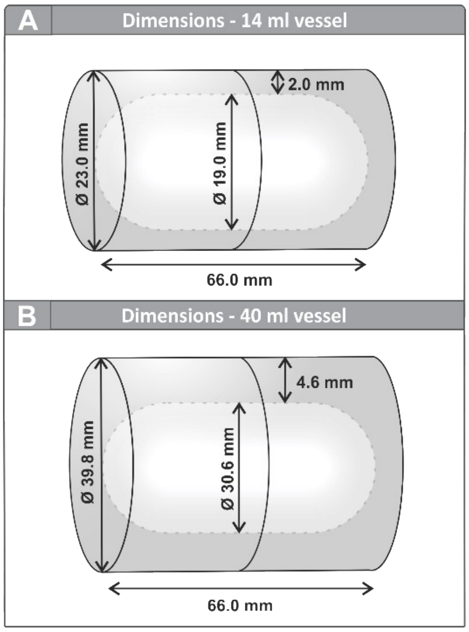

Figure S1: Figure 2: Vessel dimensions of a 14 ml vessel (A) and a 40 ml vessel (B) manufactured in the in-house tool shop.

The vessels were custom-made from the in-house toolshop through lathe processing. The solid and hollow bronze milling balls, as well as the different steel cylinders (1.3505 steel) were custom-made in the in-house toolhop as well. Bronze was selected because its superior weldability compared to the commonly used 1.3505 steel was essential for the fabrication of hollow milling balls of the investigated sizes, while the durability of the metal coating on bronze was sufficient for spherical tools. The hollow milling balls were fabricated by welding together two hemispherical bronze shells. To ensure comparability, the solid milling balls were likewise manufactured from bronze. For the cylindrical tools, 1.3505 steel was used as a precautionary measure, since coating durability on bronze proved to be lower, which could lead to increased abrasion at the sharper edges of some of the cylinders used. Table S1 is showing the geometrical parameters of the different cylinders used.

Table S1: Approximate geometrical parameters of the cylinders used. Shown are the total length of the cylinders (L_{total}), the cylinder radius (r_{total}), the lateral surface area of the rounded cylinders ($L_{lateral}$) and the radius of the rounded ends (r_{ends}). The latter corresponds to the radius of the sphere formed by the two hemispherical end caps.

**Cylinder**	L_{total} / *mm*	r_{total} / *mm*	$L_{lateral}$ / *mm*	r_{ends} / *mm*
Regular (4.6 g)	11.8	8.0		
Rounded (4.6g)	14.5	4	6.5	4
Regular (15.8 g)	26.9	7.5		
Rounded (5.8 g)	21.7	6	9.6	6

The heating setup was custom made for the used *Retsch* MM500 mixer mill (left) using heating jackets from *Ihne & Tesch* (230-250 V, 300W, CrNi-Ni), as well as a temperature control panel (right) for setting the desired temperature.

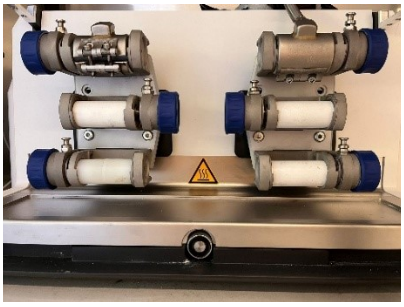

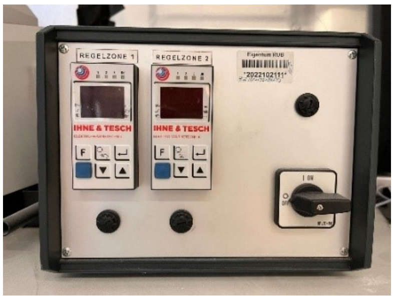

High performance liquid chromatography (HPLC) was performed on a shimadzu Nexera LC-40 lite. A Nucleodur C18, 3 µl Reversed phase column from Macherey-Nagel was used as stationary phase. An isocratic solvent mixture of 65 % acetonitrile, as well as 35 % water was used at a flowrate of 0.1 mL/min as mobile phase. The crude product (approx. 4 mg - 5 mg) was dissolved in 3 mL of the acetonitrile/water mixture (65:35) acidified with 5 % acetic acid, filtered through a syringe filter, and subsequently transferred to a HPLC vial.

Nuclear magnetic resonance spectroscopy (NMR). ^{1}H-NMR spectra were recorded in $CDCl_3$ on a Bruker Avance III HD spectrometer at 300 MHz. Spectra were referenced internally to residual solvent resonances and are reported relative to totetramethylsilane. Data are reported as follows: s = singlet, d = doublet, dd= doublet of doublet, m = multiplet; coupling constants in Hertz.

2. Synthetic procedures

2.1 Direct mechanocatalysed Suzuki coupling

For the standard reaction, phenylboronic acid (122 mg, 1.0 mmol, 1.0 eq.), potassium carbonate (1.0 g, 7.3 mmol, 7.3 eq.), 4-iodobenzaldehyde (232 mg, 1.0 mmol, 1.0 eq.), as well as *n*-butanol (311 µL, η = 0.15, 3.4 mmol, 3.4 eq.)[1,2] were place in a 14 mL vessel made of PFA or PA6. Before the reaction mixture was added, a palladium coated ball (Ø 10 mm, 15 mm) was placed inside the milling jar. The milling tools were coated according to the standard procedure developed in our group.[3] The sealed reaction vessel was shaken for 10 minutes at 35 Hz and 60 °C in a MM500 mixer mill by *Retsch*. The crude product was dissolved in water and ethyl acetate and the water phase was extracted with ethyl acetate (2x20 mL). The organic phase was dried using $MgSO_4$ and the solvent was removed under vacuum. The Product was quantified via HPLC.

2.2 Finkelstein reaction

For the standard reaction, 4-(bromomethyl)-1,1´-biphenyl (247 mg, 1.0 mmol, 1.0 eq.) and sodium iodide (449 mg, 3.0 mmol, 3.0 eq.) were placed in a 14 mL PFA vessel. Before the substrates were added, the milling ball (Ø 10 mm, 15 mm) was placed inside the milling jar. The reaction vessel was shaken for 5 minutes at 35 Hz in a MM500 mixer mill by *Retsch* at 60 °C. The crude product was dissolved in $CDCl_3$, the insoluble sodium salts were removed by filtering through a syringe filter immediately after milling to avoid aging and the yield was determined by ^{1}H NMR by the ratio of starting material to product, as there are no side reactions occuring.

3. Product characterization

4-iodomethyl-1,1´-biphenyl

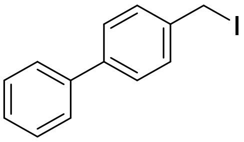

^{1}H NMR (500 MHz, $CDCl_3$) δ 7.51 (d, J = 8.6 Hz, 2H), 7.45 (d, J = 8.3 Hz, 2H), 7.39-7.35 (m, 4H), 7.28 (t, J = 7.3 Hz, 1H), 4.45 (s, 2H).

The yields shown in this study were obtained through ^{1}H NMR. Figure S2 shows an example of a spectrum measured from the product mixture.

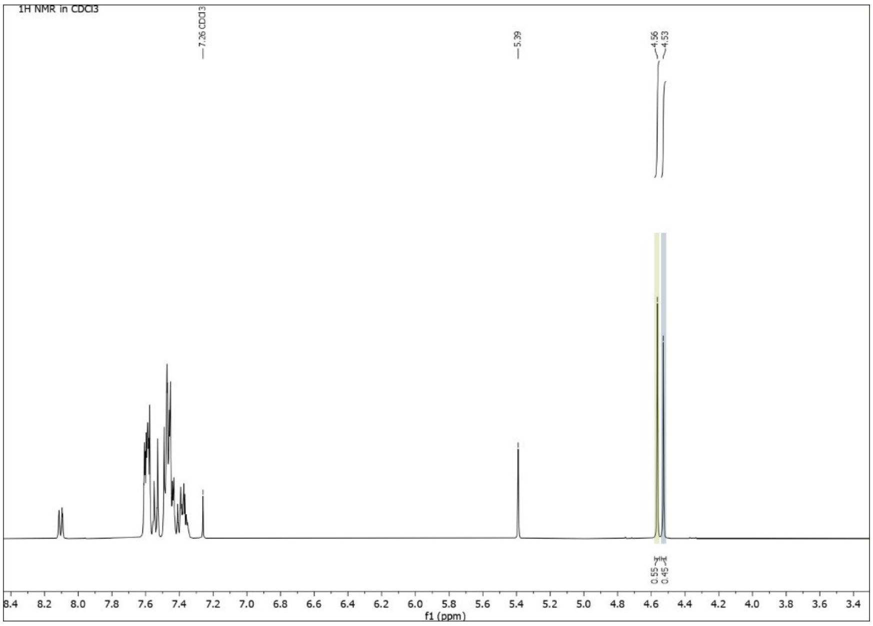

Figure S2: ^{1}H NMR spectrum of a product mixture of a Finkelstein reaction conducted in this study. The signal of 4-(bromomethyl)-1,1´-biphenyl (Substrate) is marked in green, and the Product signal is marked in blue. The signal at 5.3 ppm belongs to the standard benzyl benzoate.

Since no side reactions are possible in this case, the yield was determined from the ratio of the product and starting material peaks in the ^{1}H NMR spectra. Benzyl benzoate was used as an internal reference, as in some cases the solvent signal overlapped with the aromatic signals of both the starting material and the product.

4-biphenylcarbaldehyde

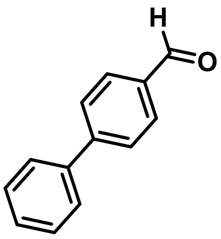

1H NMR (400 MHz, Methylene Chloride-d2) δ 10.05 (s, 1H), 8.03 – 7.91 (m, 2H), 7.82 – 7.75 (m, 2H), 7.72 – 7.63 (m, 2H), 7.49 (t, J = 7.5 Hz, 2H), 7.46 – 7.38 (m, 1H)

4. Kinetic energy calculations

The kinetic energies reported in this work were determined in our previous study.[4] A detailed description of their determination is provided in the following paragraphs. A schematic representation of the set up used for high-speed recordings is shown in Figure S3.

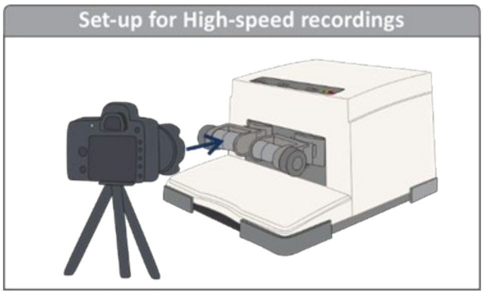

Figure S3: Schematic representation of the setup used for conducting the high-speed recordings. When referring to "the forward-facing wall", this denotes the wall facing the camera.

The trajectories of the milling balls were analysed using the open-source software *Tracker Video Analysis and Modeling Tool*. High-speed recordings were imported at the original frame rate of the respective experiments (1000 frames per second (fps)). A known reference length – the outer length of the milling vessel – was used for spatial calibration within the software. The position of each milling ball was determined manually frame-by-frame over sequences of 500–1500 frames, depending on the visibility of the ball in the respective recording.

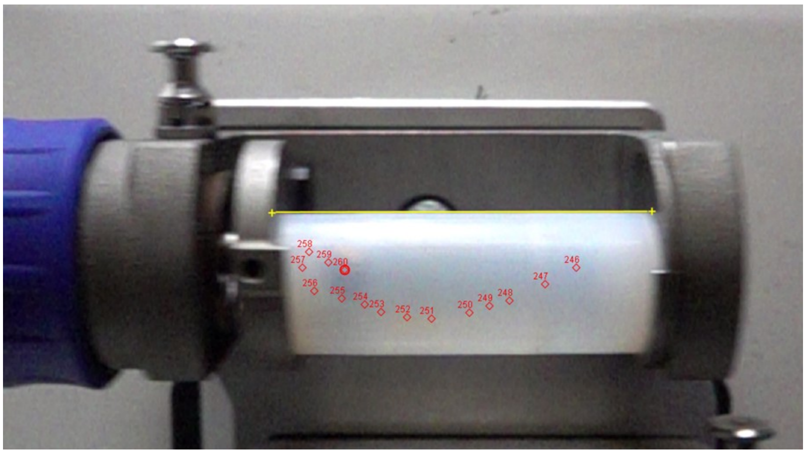

Figure S4: A frame from the *Tracker Video Analysis and Modeling Tool* illustrating the tracking of the ball position. The yellow line, corresponding to the known vessel length, was used as a reference to convert pixel distances into real distances. The ball center was manually marked in each frame, as the semi-translucent vessel limited visibility and prevented automatic tracking.

The calibrated x and y coordinates were exported and processed to calculate the displacement per frame. From these displacements and the known frame rate, the instantaneous velocities were obtained. For each trajectory, the mean velocity over the complete tracked sequence was calculated and used to determine the kinetic energy:

$$E_{kin} = \frac{1}{2} m \bar{v}^2 \tag{1}$$

where m is the mass of the respective milling ball and $\bar{v}$ the mean velocity. This approach reduces the influence of single-frame deviations or tracking inaccuracies and yields a representative kinetic energy value for the observed motion. In cases where the ball's motion included rolling components, this contribution was implicitly included in the measured displacement values.

It should be noted, however, that these measurements are not fully precise, as variations in the inclination of the holder may have slightly shortened the measured distances. Furthermore, certain points located at the ends of the vessel can be difficult to detect, as the PFA vessel used is only semi-transparent, which may have resulted in slight deviations as well. Additionally, the movement in the z-direction (along the viewing axis of the camera) cannot be observed, which may further account for slight differences of the kinetic energies shown. The kinetic energies calculated for the cylindrical milling tools may deviate

from the presented results, since it is not always possible to strike the center of the cylinders due to their asymmetry and irregular motion.

5. Hollow vs solid milling balls – Role of mass and surface area

5.1 Suzuki coupling

5.1.1 Suzuki coupling PA vessel vs. PFA vessel

For the Suzuki coupling of 4-iodobenzaldehyde and phenylboronic acid using hollow and solid milling balls of different sizes, a 14 mL polyamide 6 (PA 6) vessel was used, as the previously used PFA vessel was not sealed inside the used heating jacket. As the other reactions, as well as the high-speed recordings were conducted using a PFA vessel, the question arises whether the results are comparably high for both vessel types. Figure S5 shows the results for a Suzuki coupling using both vessel materials.

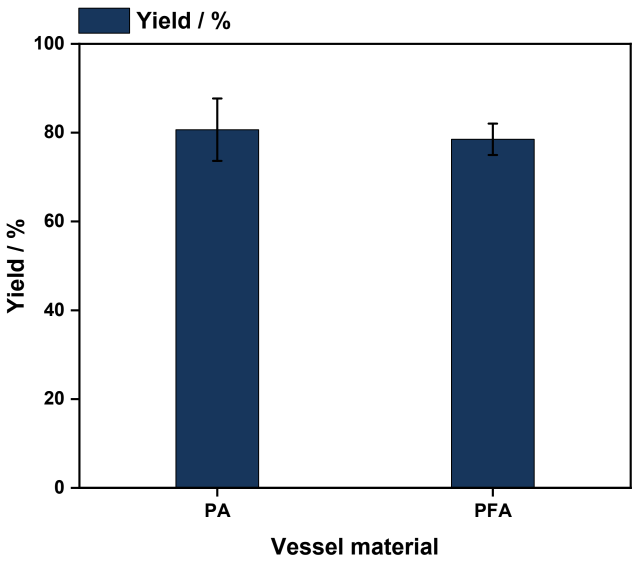

Figure S5: Yields of the Suzuki coupling with a PFA vessel compared to a PA 6 vessel. The experiments were performed in a MM500 mixer mill by *Retsch* at a milling frequency of 35 Hz for 10 minutes at 60°C. A solid 10 mm milling ball and the reaction mixture (1 g K_2CO_3, 122 mg phenylboronic acid, 232 mg iodobenzene, 311 µL *n*-Butanol) were added to the respective vessel before milling.

It can be observed that both vessel materials show similar results. Furthermore, a quantitative comparison of the results of the different screenings was not done in this study, further showing that this procedure was applicable in this case.

5.1.2 Suzuki Coupling – 14 mL vessel

It is of particular interest to observe, what role the mass and surface area of a milling ball play in mechanochemistry, but especially in direct mechanocatalysis. For this reason, solid and hollow milling balls were used to be able to decouple mass and surface area. This has been compared at different milling frequencies for 10 mm and 15 mm solid and hollow milling balls. Figure S6 shows the yields, as well as the yields normalized to the mass at different milling frequencies (30Hz 25 Hz, 20 Hz).

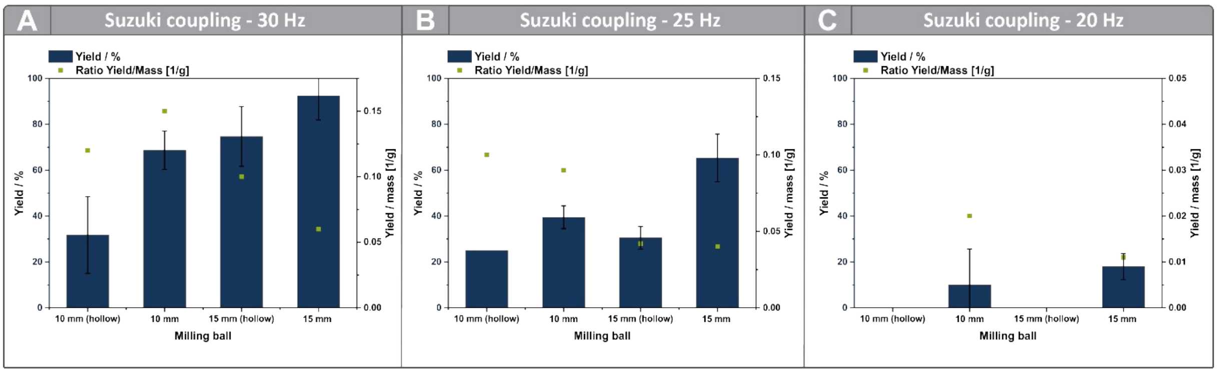

Figure S6: Yields of the Suzuki coupling reaction, as well as the ratio of yield and mass of the different solid and hollow milling balls utilized (Ø 10 mm and 15 mm). The experiments were performed in a MM500 mixer mill by *Retsch* at a milling frequency of 30 Hz (A), 25 Hz (B) and 20 Hz (C) for 10 minutes at 60°C. A 14 mL PA 6 vessel containing the milling material and the reaction mixture (1 g K_2CO_3, 122 mg phenylboronic acid, 232 mg iodobenzene, 311 µL *n*-Butanol) was utilized.

At 30 Hz, the yield normalized per gram of milling ball does not decrease linearly. This deviation is primarily due to the significantly different yields obtained with the hollow 10 mm ball. As a result, the mean yield is likely lower, and the trend does not match the one observed at 35 Hz. The variation in yields for this ball could be attributed to human error during handling or measurement, as well as possible defects in the palladium coating. For the other investigated frequencies, the decrease in yield per gram of milling ball is approximately linear. This supports the conclusion that hollow balls are generally more efficient than solid ones being able to move in a figure-eight manner, and that larger balls tend to be less efficient than smaller balls due to the less preferred flight path. The deviations from the linear trend observed at lower frequencies can furthermore be explained by the increased deflection from the balls' preferred trajectory compared to the stable motion patterns seen at 35 Hz.

5.1.3 Suzuki coupling – 40 mL vessel

As the comparison between the solid 15 mm and hollow 20 mm balls is of primary interest, the other two balls were not included in the main manuscript. Figure S7, however, presents the results for all four balls used in this study at 35 Hz. Similar to the observations in the smaller vessel, the hollow balls show higher efficiency when considering the normalized yields relative to the mass and kinetic energy of the milling ball. For the yields normalized to the total surface area of the milling ball, these balls are also showing similar trends compared to the smaller scale approach. Further details on the comparison between the solid 15 mm and hollow 20 mm balls can be found in the main manuscript.

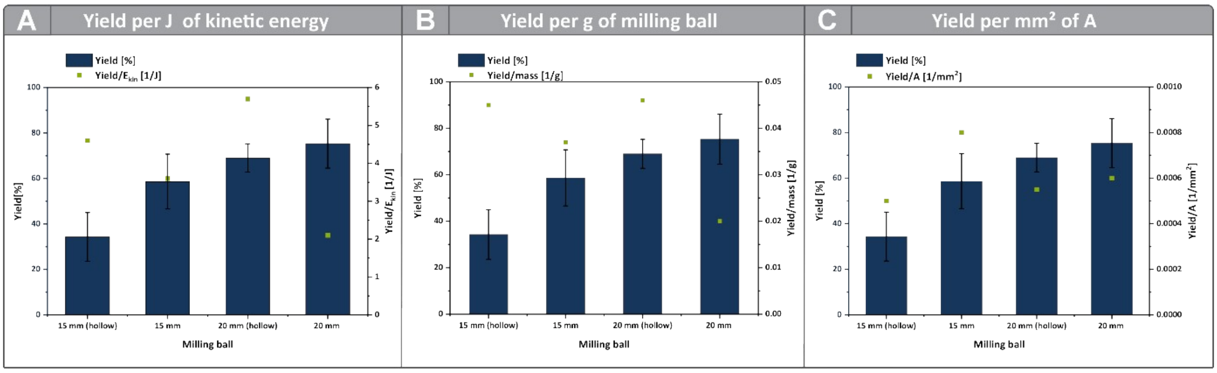

Figure S7: Yields of the Suzuki coupling reaction, as well as the ratio of yield and kinetic energy (A), mass (B) and surface area (C) of the different solid and hollow milling balls utilized (Ø 15 mm and 20 mm). The experiments were performed in a MM500 mixer mill by *Retsch* at a milling frequency of 35 Hz for 5 minutes at 60°C. A 40 mL PFA vessel containing the milling material and the reaction mixture (2.8 g K_2CO_3, 349 mg phenylboronic acid, 663 mg iodobenzene, 888 µL *n*-Butanol) was utilized.

The shown screening in chapter 5.1.1 was repeated for the larger vessel type. Figure S8 shows the Yields, as well as the yields normalized per gram of milling ball for the different sizes hollow and solid milling balls at frequencies of 30 Hz, 25 Hz and 20 Hz.

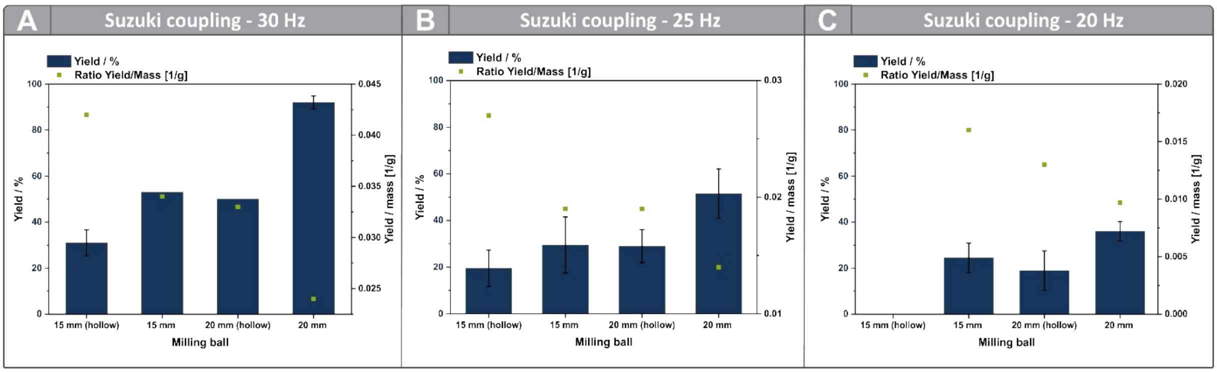

Figure S8: Yields of the Suzuki coupling reaction, as well as the ratio of yield and mass of the different solid and hollow milling balls utilized (Ø 15 mm and 20 mm). The experiments were performed in a MM500 mixer mill by *Retsch* at a milling frequency of 30 Hz (A), 25 Hz (B) and 20 Hz (C) for 5 minutes at 60°C. A 40 mL PFA vessel containing the milling material and the reaction mixture (2.8 g K_2CO_3, 348 mg phenylboronic acid, 662 mg iodobenzene, 888 µl *n*-Butanol) was utilized.

For 30 Hz and 25 Hz, the yields per gram of milling ball approximately follow the trend observed at 35 Hz. At 20 Hz, however, the yield per gram of the hollow 20 mm ball is higher than that of the solid 15 mm ball. This deviation could be explained by the reduced contact frequency between the balls and the vessel walls, as well as the more unpredictable ball motion, which may have contributed to the altered trend.

5.2 Finkelstein reaction

The following figure shows the yields, as well as the yields normalized per gram of milling ball for the Finkelstein reaction at 25 Hz.

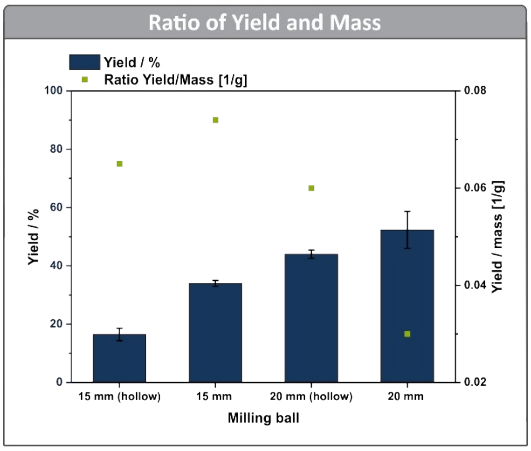

Figure S9: Yields of the Finkelstein reaction, as well as the ratio of yield and mass of the different milling balls utilized (Ø 10 mm and 15 mm). The experiments were performed in a MM500 mixer mill by *Retsch* at a milling frequency of 25 Hz for 5 minutes at 60°C. A 14 mL PFA vessel containing the milling tool and the reaction mixture (247 mg 4-(bromomethyl)-1,1´-biphenyl and 449 mg sodium iodide) was utilized.

In this case, the yield per gram of milling ball is lower for the hollow 10 mm ball compared to its solid equivalent. This might be due to the increased deviation from the figure-eight shaped movement, which might be higher through the low mass and reduced frequency.

References

1 J.-L. Do and T. Friščić, Mechanochemistry: A Force of Synthesis, *ACS Cent. Sci.*, 2017, **3**, 13–19.

2 W. Pickhardt, C. Beaković, M. Mayer, M. Wohlgemuth, F. J. L. Kraus, M. Etter, S. Grätz and L. Borchardt, The Direct Mechanocatalytic Suzuki-Miyaura Reaction of Small Organic Molecules, *Angew. Chem., Int. Ed.*, 2022, **61**, e202205003.

3 M. Wohlgemuth, M. Mayer, M. Rappen, F. Schmidt, R. Saure, S. Grätz and L. Borchardt, From Inert to Catalytically Active Milling Media: Galvanostatic Coating for Direct Mechanocatalysis, *Angew. Chem., Int. Ed.*, 2022, **61**, e202212694.

4 M. F. Rappen, J. Mäder, S. Grätz and L. Borchardt, Motion matters: the role of milling ball trajectories in mechanochemical reactions, *RSC Mechanochem.*, 2025. DOI: 10.1039/D5MR00112A.
